# Is Apomorphine a Safe Emetic?

**Published:** 1889-07

**Authors:** John Brown


### IS APOMORPHINE A SAFE EMETIC?

By John Brown, M. B., B. Ch. Vict., etc.

Recently the above query has appeared in the *Journal.* I wrote an article on "Apomorphine, a Safe, Certain and Quick Emetic," which appeared in the *Journal* for May 12th, 1883, p. 907. It occurred to me that it might be of interest to those correspondents and others if I gave a brief account of my further experience of this most valuable emetic. There are few, if any, of the new drugs introduced into the *British Pharmacopœia* which have sustained the high opinion of their therapeutical value with such unvarying success and with so few failures as apomorphine.

The following ten cases *seriatim*. I prepare my own solution of apomorphine as follows: Apomorphin. chlorid., gr. j.; sp. vini rect. m. 20; aquæ m. 110. Each 10 minims equals one twelfth of a grain of the alkaloid.

Case 1. A. B., aged about 30; 10 minims administered hypodermically was followed in five minutes by free and copious vomiting. No bad after-effects.

Case 2. J. H., aged 5 years, swallowed ammonia; 2 minims were given; in three minutes afterwards there was free emesis. The child did remarkably well.

Case 3. J. J., aged 4 years, swallowed a clay pipe. Dr. S. sent for me to give apomorphine, as other emetics had failed, I gave 3 minims, and in six minutes, without any previous nausea, vomiting followed.

Case 4. A. E. B., aged 2 years, supposed to have swallowed a farthing; 2 minims were administered; this was followed in six minutes by vomiting.

Case 5. C. M. B., aged 14 months, swallowed a button; 2 minims were given; in six minutes copious vomiting followed.

Case 6. Mrs. M., aged 24, suffering from most violent spasms of laryngeal muscles due to irritation of the stomach from a mass of indigestible food; 10 minims were given, and produced very free vomiting, which was repeated two or three times. The result was speedy relief of the spasms; no prostration or collapse.

Case 7. H. M., aged 4 years, was supposed to have swallowed a quantity of indigestible fruit; 3 minims were given, and in eleven minutes free emesis followed.

Case 8. W. C., aged 3½ years, suffering from croup; 2½ minims were given, and in fourteen minutes free emesis followed.

Case 9. D. L., aged 2½ years; gave two minims; a small portion oozed out from where the needle was inserted, and vomiting did not follow for twenty-eight minutes, which is the longest interval I have ever observed, probably owing to the smallness of the dose, which might have been from $\frac{1}{100}$ to $\frac{1}{120}$ of a grain of the alkaloid.

Case 10. Miss M., aged 4 years; 4 minims were given and were followed by vomiting in eighteen minutes.

The average interval between the hypodermic injection and the emesis is 10.1 minutes. As a rule, the vomiting only occurs two or three times at short intervals. The depression is only what might be expected after an ordinary vomiting. I have observed no case approaching fatal or even serious collapse. Only two of the cases were adults, the others were very young children. Cases 4 and 5 were my own children, and I have the greatest confidence in giving it hypodermically to any of my children who may require an emetic. Every one must have observed how difficult it is to make an infant or any child under 4 years vomit by giving emetics by the mouth. I have been surprised that authors on children's diseases do not recommend apomorphine hypodermically. Dr. Angel Money's work on *Treatment of Diseases in Children* is very excellent, but I find no mention of apomorphine as an emetic. I believe there is no emetic "so safe, certain and quick" for children. In adults ordinary emetics usually succeed; not so in children. The cases reported in which collapse occurred were adults. It may be that apomorphine may occasionally cause collapse—although I have not observed any case—in adults, yet be perfectly safe for children, similar to the action of chloroform in adults and in children. Apomorphine is the emetic *par excellence* for children when given hypodermically.—*British Medical Journal.*

